# Systemic Parathyroid Hormone Enhances Fracture Healing in Multiple Murine Models of Type 2 Diabetes Mellitus

**DOI:** 10.1002/jbm4.10359

**Published:** 2020-04-02

**Authors:** Kareme D Alder, Andrew HA White, Yeon‐Ho Chung, Inkyu Lee, JungHo Back, Hyuk‐Kwon Kwon, Sean V Cahill, Zichen Hao, Lu Li, Fancheng Chen, Saelim Lee, Matthew D Riedel, Francis Y Lee

**Affiliations:** ^1^ Department of Orthopædics & Rehabilitation Yale University, School of Medicine New Haven CT USA; ^2^ Department of Life Science Chung‐Ang University Seoul Republic of Korea

**Keywords:** DIABETIC OSTEOPOROSIS, FRACTURE HEALING, ORTHOPEDIC TRAUMA, PARATHYROID HORMONE, TYPE 2 DIABETES

## Abstract

Type 2 diabetes mellitus (T2DM) is a multisystemic disease that afflicts more than 415 million people globally—the incidence and prevalence of T2DM continues to rise. It is well‐known that T2DM has detrimental effects on bone quality that increase skeletal fragility, which predisposes subjects to an increased risk of fracture and fracture healing that results in non‐ or malunion. Diabetics have been found to have perturbations in metabolism, hormone production, and calcium homeostasis—particularly PTH expression—that contribute to the increased risk of fracture and decreased fracture healing. Given the perturbations in PTH expression and the establishment of hPTH (1–34) for use in age‐related osteoporosis, it was determined logical to attempt to ameliorate the bone phenotype found in T2DM using hPTH (1–34). Therefore, the present study had two aims: (i) to establish a suitable murine model of the skeletal fragility present in T2DM because no current consensus model exists; and (ii) to determine the effects of hPTH (1–34) on bone fractures in T2DM. The results of the present study suggest that the polygenic mouse of T2DM, TALLYHO/JngJ, most accurately recapitulates the diabetic osteoporotic phenotype seen in humans and that the intermittent systemic administration of hPTH (1–34) increases fracture healing in T2DM murine models by increasing the proliferation of mesenchymal stem cells. © 2020 The Authors. *JBMR Plus* published by Wiley Periodicals, Inc. on behalf of American Society for Bone and Mineral Research.

## Introduction

PTH, an 84‐amino acid peptide released by the parathyroid glands, is an important calcium‐regulating hormone that aids in regulating bone remodeling. PTH is released from the parathyroid glands in response to hypocalcemic states and increases serum calcium levels by stimulating the production of 1,25‐dihydroxyvitamin D [1,25(OH)_2_D], which in turn increases gastrointestinal calcium absorption, renal calcium reabsorption, and the rate of bone resorption by osteoclasts.^1^ Although the sustained increase in PTH secretion seen in hyperparathyroidism results in bone loss caused by an upregulation of osteoclastic bone resorption, transient rises in PTH levels have been shown to increase bone mass as PTH's anabolic properties outweigh its catabolic activity when used in this modality.^2^ Indeed, multiple animal studies have shown that brief increases in PTH levels stimulate endochondral bone formation and promote mesenchymal stem cell (MSC) differentiation into osteoblasts.^1^, ^3^, ^4^ Additionally, intermittent s.c. administration of PTH has been shown to increase the load properties and mineral content in fractures.^5^ Currently, daily s.c. injections of recombinant human PTH amino‐terminal peptide 1–34 [hPTH (1–34)], or the full‐length protein, hPTH (1–84), are used to treat osteoporosis in human patients.^6^

Multiple studies have shown that patients with type 2 diabetes mellitus (T2DM) are more likely to suffer osteoporotic fractures than nondiabetics.^7^, ^8^, ^9^ Undeniably, such fractures are a debilitating complication of diabetes that impairs quality of life in millions of people globally. This also leads to the expenditure of billions of health care dollars in the United States caused by the direct care and the loss of productivity of patients from complications that follow fractures.^10^, ^11^, ^12^, ^13^, ^14^ Unfortunately, the rate of T2DM and its associated fractures continues to rise in the United States. As of 2017, the Centers for Disease Control and Prevention reports that 23.1 million Americans, or 7.2% of the population, have diagnosed diabetes, with only 5% being T1DM.^15^ When they include estimates for those undiagnosed, this number increases to 30.3 million, or 9.4% of the population. These figures are in addition to the 84.1 million Americans 18 years of age or older who have prediabetes and are at risk of developing the disease in the next 5 years.^15^

The exact connection between diabetic pathophysiology and an increased risk of bone fracture is unknown. Hyperglycemic states have been shown to cause an increase in urinary calcium loss, resulting in a negative calcium balance that may contribute to the increased rate of bone fractures in diabetic patients.^7^ Perhaps more significantly, the persistent inflammatory state seen with chronic hyperglycemia increases chondrocyte and osteoblast apoptosis, while simultaneously encouraging osteoclast survival, tipping the scales of bone remodeling in favor of bone loss.^16^ Similarly, inflammatory markers, including TNFα, inhibit angiogenesis, a vital component of fracture healing and bone health.^17^ This state of calcium depletion, remodeling inequality, and angiogenic insufficiency may contribute both to the increased fracture risk and the delayed fracture healing seen in diabetic populations.

T2DM has a diverse phenotype stemming from many etiologies in humans because genetics and environment both play a significant role in the development of the disease. Further, T2DM is associated with many comorbidities, particularly obesity, dyslipidemia, and hypertension, a trend that holds true even in adolescent populations.^18^ As such, there is no single mouse model that fully encompasses all the varied features of clinical T2DM. The present study utilizes two distinct murine models of T2DM. The first murine model, B6.BKS(D)‐Lepr^db^/J (db/db), has a monogenetic knockout mutation in the leptin receptor gene resulting in hyperphagia, marked obesity, and consequent hyperglycemia. The second murine model, TALLYHO/JngJ, is a polygenetic model of T2DM that accurately reflects the many characteristic phenotypes of human T2DM. Together, these models cover a wide variety of T2DM etiologies and offer insight into the human disease.

With these chosen models of T2DM, the current study investigates: (i) which murine model of skeletal fragility found in T2DM is most representative of the human phenotype; and (ii) the potential use of systemic hPTH (1–34) in diabetic fracture healing. Intermittent systemic treatment of hPTH (1–34) has previously been successful in improving bone quality for osteoporotic human patients.^6^ Moreover, diabetes is associated with increased urinary calcium excretion, and although the associated low levels of serum calcium usually induce an increase in parathyroid activity, PTH levels in patients with DM have been shown to be 55% of normal.^19^ Of note, low PTH secretion is itself a cause of poor bone quality in T2DM patients, and is not suggestive of resistance to PTH at the osteoblast level.^20^ This functional hypoparathyroidism makes PTH an area of interest in diabetic bone research and identifies T2DM patients with fractures as ideal candidates for potential treatment with exogenous PTH.

The data from the present study suggest that the monogenic murine model db/db is the most accurate representation of human diabetic osteoporosis and that the intermittent administration of hPTH (1–34) may improve fracture healing capacity in human patients with T2DM as evidenced by this effect in the chosen murine models.

## Materials and Methods

### Murine models

All animal experiment protocols were approved by Yale University School of Medicine's Institutional Animal Care and Use Committee. Up to five mice were housed in sterile, ventilated cages and were allowed food and water *ad libitum* within Yale University School of Medicine's animal facility. Chow was provided by Yale University. Male, 10‐week‐old C57BL/6J (Stock No: 000664), B6.BKS(D)‐Lepr^db^/J (db/db) (Stock No: 000697), Swiss Webster/J (SWR/J) (Stock No: 000689), and TALLYHO/JngJ (Stock No: 005314) strains were all purchased from the Jackson Laboratory (Bar Harbor, ME, USA). Animals were randomized to treatment and control groups; researchers were not blinded. No important adverse events occurred during animal experiments.

### Blood glucose measurements

All blood glucose measurements were obtained using a commercially available glucometer and test strips (One Touch Ultra 2; LifeScan, Inc., Milpitas, CA, USA) after fasting the mice for 16 hours. Blood was obtained from the tail vein following anesthesia.

### Surgical method

**Figure 1:**
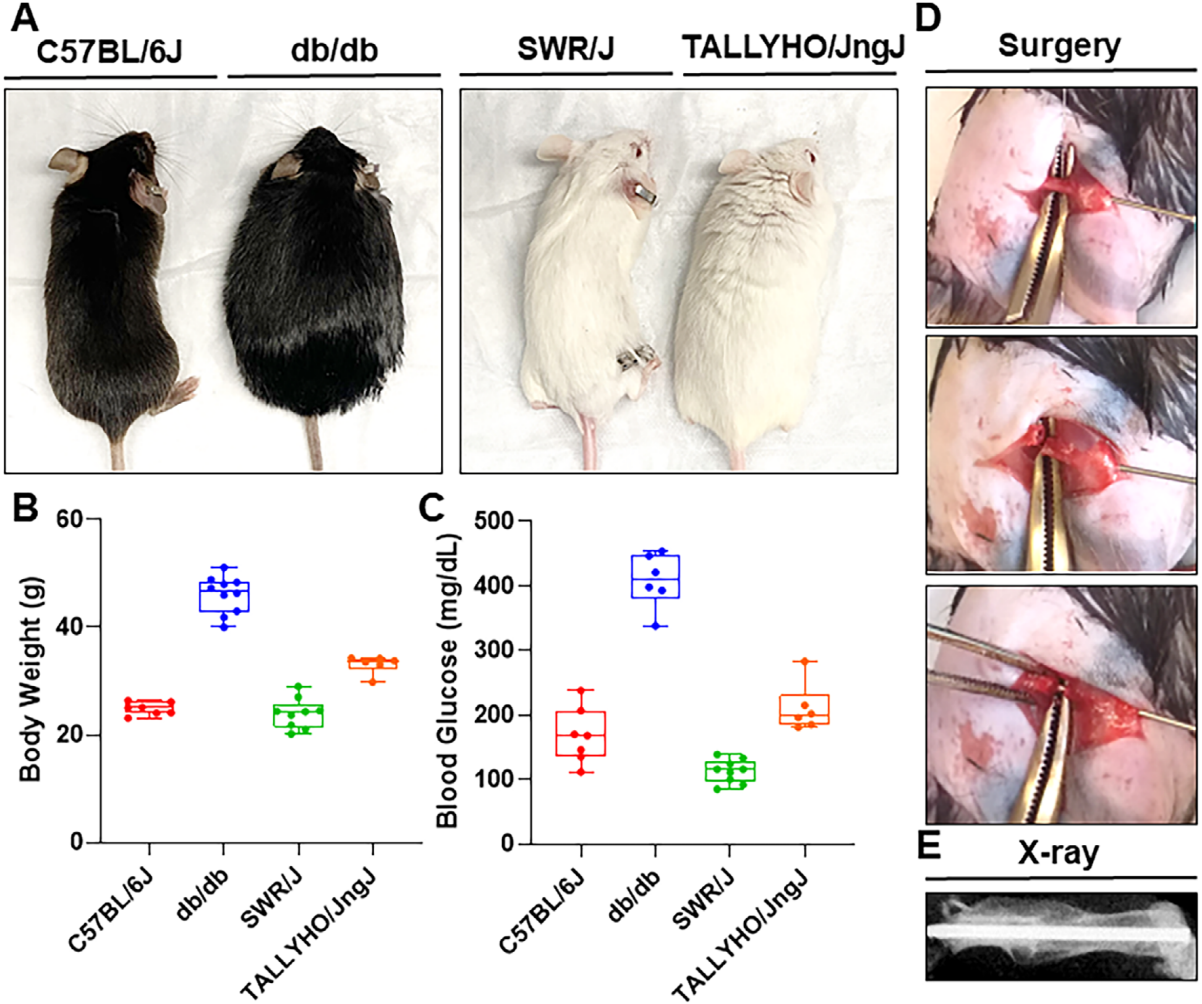
Body mass, blood glucose measurements, and surgical technique in type 2 diabetes mellitus murine models. (*A*) Gross body weight differences in murine strains. (*B*) Body weight (g) of each murine strain was quantified using an analytical scale. C57BL/6J models showed an average body weight of 24.8 ± 1.2 g (*n* = 6), whereas the db/db model average body weight was 46.1 ± 3.5 g (*n* = 6). The SWR/J model average body weight was 24.0 ± 2.8 g (*n* = 9), whereas TALLYHO/JngJ models had an average body weight of 33.1 ± 1.7 g (*n* = 6). (*C*) Blood glucose measurements (mg/dL) of murine models were taken using a glucometer (One Touch Ultra 2; LifeScan, Inc., Milpitas, CA, USA) after 16 hours of fasting. C57BL/6J mice showed average blood glucose levels of 148.0 ± 43.1 mg/dL (*n* = 6); db/db mice showed average glucose levels of 408.5 ± 42.5 mg/dL (*n* = 6). SWR/J showed average glucose levels of 112.9 ± 18.1 mg/dL (*n* = 9); TALLYHO/JngJ showed average glucose levels of 210.3 ± 37.6 mg/dL (*n* = 6). Body weights and fasting plasma glucose levels of all mice were measured at 10 weeks of age. (*D*) Key steps during surgery: placement of Gigli saw wire for fracture induction (upper panel), induction of transverse fracture (middle panel), and fixation of fracture (lower panel). (*E*) X‐ray image verifying correct pin placement after surgery.

A ketamine (Zoetis Inc., Kalamazoo, MI, USA) / xylazine solution (Akorn Inc., Lake Forest, IL, USA) was administered i.p. at a dose of 100 mg/kg / 10 mg/kg to induce anesthesia. The depth of anesthesia was assessed by response to tail, pinnae, or pedal pinch. Preemptively, buprenorphine (0.05 mg/kg i.p.) was administered for general pain control, whereas the surgical incision was infiltrated with bupivacaine (AuroMedics Pharma LLC, E. Windsor, NJ, USA) for local pain control (4 mg/kg s.q.). An incision extending from the hip to the knee joint was made on the lateral right hindlimb. The patella was displaced laterally, and a 1‐inch 23‐gauge needle was inserted into the intramedullary space along the entire length of the femur from the femoral condyles to the femoral neck. The lateral thigh muscles were spread apart bluntly to expose the femoral shaft, and a length of Gigli saw wire was passed around the femur with care to protect the soft tissues and neurovascular structures (Fig. 1 *D*, upper panel). The intramedullary needle was then withdrawn halfway, and the Gigli saw wire was used to create a transverse midshaft fracture in the femur (Fig. 1 *D*, middle panel). The needle was then readvanced past the created fracture into the proximal femur and the musculature was returned to anatomical position (Fig. 1 *D*, lower panel). The needle was then clipped at the end of the distal femur, leaving a length of needle in the intramedullary space. The patella was returned to anatomical position; the musculature was opposed using suture; and the skin incision was closed with surgical staples. hPTH (1–34) (40 μg/kg; Alfa Aesar, Haverhill, MA, USA) was injected s.c. immediately following surgery for mice in the treatment group. Analgesic (buprenorphine; Reckitt Benckiser Healthcare, Hull, England) and antibiotic (10 mg/kg enrofloxacin; Bayer, Leverkusen, Germany) treatments were maintained for 72 hours following surgery. X‐ray was used to verify pin placement (Fig. 1 *E*).

### μCT studies

μCT analysis of femurs was performed in 10‐week‐old mice for baseline studies and at 28 days postfracture for fracture healing studies. An 11.5‐mm sample of the femoral shaft was used for mineralized callus μCT analysis on a Scanco μCT 35 system (Scanco Medical, Brüttisellen, Switzerland). A 10‐μm voxel size, 55 KVp, 0.36‐degree rotation step (180‐degree angular range), and a 400‐ms exposure per view were used for the scans that were performed in PBS solution. The Scanco μCT software (HP, DECwindows Motif 1.6) was used for 3D reconstruction and viewing of images. After 3D reconstruction, callus volumes with extracted pre‐existing cortex were segmented using a global threshold of 0.4 g/c. Tissue mineral density (TMD), direct total volume (TV), bone volume (BV), and bone volume fraction (BV/TV) were calculated for each callus. Starting 100 μm from the growth plate, 1.35 mm of the distal part of each femur and a 1.4 mm part of the mid‐diaphysis were used for trabecular and cortical bone (respectively) μCT analysis on the Scanco μCT 35 system, as described above. Relative cortical area (Ct.Ar/Tt.Ar), thickness of the cortex (Ct.Th), and polar moment of inertia were calculated for cortical bone. TMD, apparent density, and directly measured bone volume fraction (BV/TV), surface‐to‐volume ratio (BS/BV), trabecular thickness (Tb.Th), trabecular number (Tb.N), and trabecular separation (Tb.Sp) were calculated for the trabecular bone.

### Histology and histomorphometry

Harvested femora were fixed for 1 day in 4% paraformaldehyde and PBS directly after euthanasia. After fixation, the bones were rinsed and decalcified with 10% EDTA (pH 7.2 to 7.4) for 2 weeks on a shaker. The intramedullary pins were removed from the bones before embedding and sectioning. After, the specimens were processed to a thickness of 5 μm. Immunohistochemistry (IHC) was performed using the MACH 4 Universal HRP‐Polymer (Biocare Medical, LLC, Pacheco, CA, USA) to detect anti‐CD51 mouse antibody (MAI‐33578; Abcam, Cambridge, MA, USA) and anti‐PPARɤ rabbit monoclonal antibody (2435; Cell Signaling Technology, Inc., Danvers, MA, USA). Reactions were then visualized with diaminobenzidine (DAB) as substrate. The sections were counterstained with hematoxylin. All staining procedures were followed as instructed by the manufacturer. ImageJ software (NIH, Bethesda, MD, USA; https://imagej.nih.gov/ij/) was used to count the number of positive stained cells. Additionally, multiplex immunohistochemistry (m‐IHC) was performed using fluorescein, cyanine 3, and cyanine 5 dyes (PerkinElmer, Boston, MA, USA) for m‐IHC according to the manufacturer's instructions. Lastly, cartilage was defined as tissue with positive safranin O/fast green staining. Images of histologic sections were captured using a Cytation 5 Imaging Reader (Biotek, Winooski, VT, USA). ImageJ software was used to count the number of pixels for analysis.

### Bone mechanical strength testing

After scanning, the specimen was mounted on a three‐point‐bending jig on an electromagnetic loading machine. The loading location was determined at 55% of the femur length from the femoral head. Dynamic mechanical analysis (DMA) was performed by applying nondestructive compressive oscillatory displacement with a mean level of 0.01 mm and amplitude of 0.005 mm at the range of 0.5 to 3 Hz. A displacement transducer with 15‐nm resolution was utilized to control displacement and detect the small magnitude of signal changes. Dynamic complex stiffness (K*) was computed from elastic (storage; K′) and viscous (loss; K′′) stiffness with an equation of K* = K′ + iK′′ using the cyclic force and displacement. Tangent delta (tan δ), which accounts for ability of loading energy dissipation, was computed by K′′/K′. Following the nondestructive DMA testing, static fracture testing was conducted with a displacement rate of 0.5 mm/s up to fracture. Static stiffness was measured using the maximum slope of force‐displacement curve and maximum force was assessed at the fracture point.

### Statistical analysis

All analyses were performed using GraphPad Prism (GraphPad Software, Inc., La Jolla, CA, USA). Data were analyzed using one‐way ANOVA and Tukey post hoc correction. Statistical significance was assigned for *p* ≤ 0.05. All experiments were done in triplicate.

## Results

### T2DM murine models recapitulate the human T2DM phenotype

Body weight data showed experimental mouse models, db/db and TALLYHO/JngJ, to have increased body mass as compared with their control strains, C57BL/6J and SWR/J (Fig. 1 *A*,*B*). In comparison with C57BL/6J mice that have an average body weight of 24.8 ± 1.2 g, db/db mice had an average body weight of 46.1 ± 3.5 g. Also, in comparison with SWR/J, which had an average body mass of 24.0 ± 2.8 g, TALLYHO/JngJ mice had an elevated average weight of 33.1 ± 1.7 g. Moreover, the current study found hyperglycemia in both experimental mouse strains by measuring baseline blood glucose levels in each mouse group after 16 hours of fasting. Both db/db and TALLYHO/JngJ mice had elevated average blood glucose levels of 408.5 ± 42.5 mg/dL and 210.3 ± 37.6 mg/dL as compared with control strains C57BL/6J and SWR/J, which had average blood glucose levels of 148.0 ± 43.1 mg/dL and 112.9 ± 18.1 mg/dL, respectively (Fig. 1 *C*).

### μCT analysis of femora from T2DM murine models

**Figure 2:**
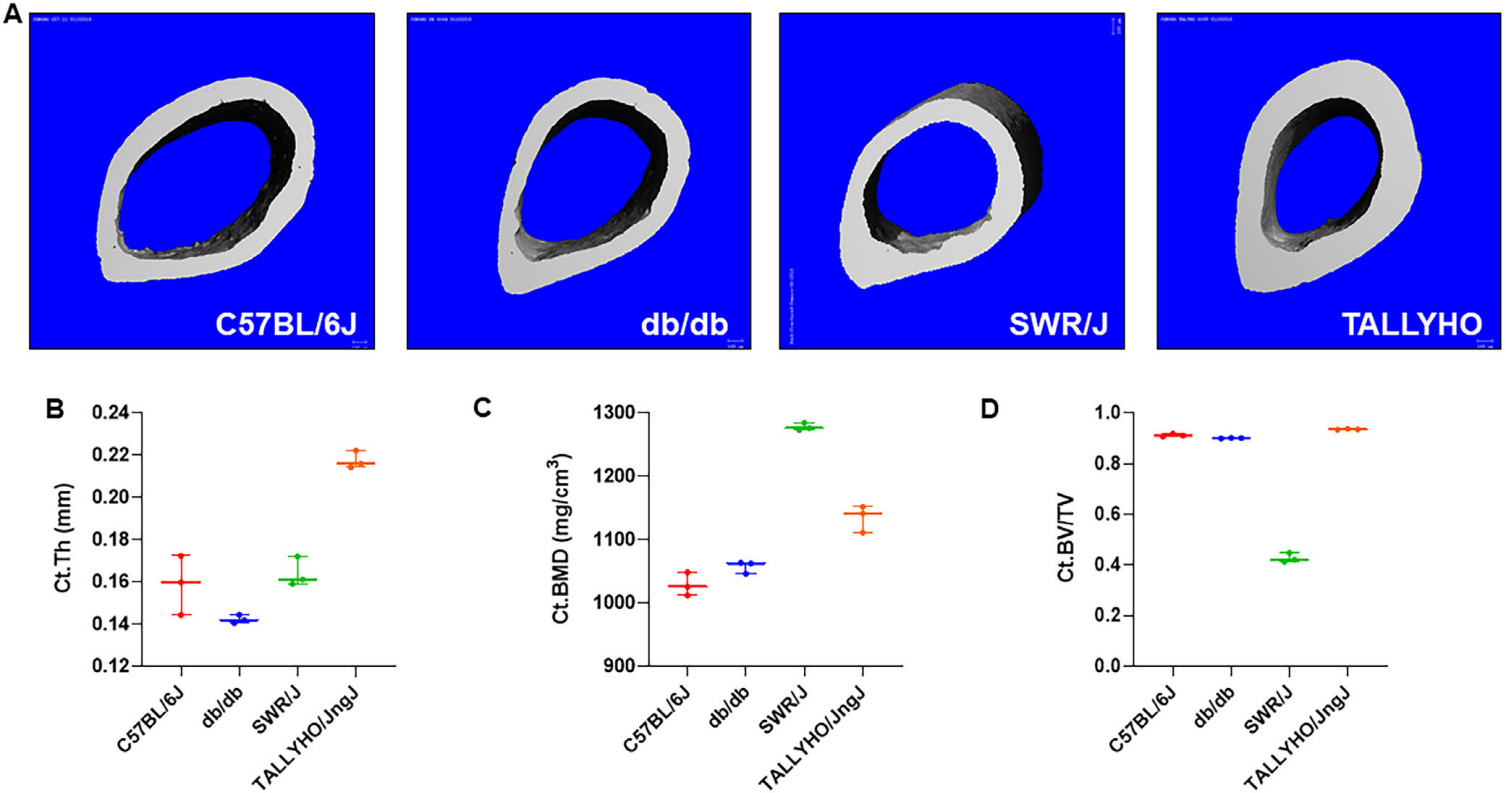
μCT analysis of femoral cortical bone from type 2 diabetes mellitus murine models. (*A*) Representative images of the cross sections of the mid‐diaphysis of murine femurs. (*B–D*) Untreated femora from 10‐week‐old mice were analyzed using a Scanco μCT 35 system (Scanco Medical, Brüttisellen, Switzerland). C57BL/6J mice (*n* = 3) showed an average cortical thickness (Ct.Th) of 0.16 ± 0.01 mm, cortical bone mineral density (Ct.BMD) of 1028.7 ± 18.6 mg/cm^3^, and cortical bone volume fraction (Ct.BV/TV) of 0.91 ± 0.006. Db/db mice (*n* = 3) showed an average Ct.Th of 0.14 ± 0.002 mm, Ct.BMD was 1057.4 ± 9.8 mg/cm^3^, and Ct.BV/TV was 0.90 ± 0.001. SWR/J mice (*n* = 3) showed an average Ct.Th of 0.16 ± 0.007 mm, Ct.BMD of 1278.0 ± 5.6 mg/cm^3^, and Ct.BV/TV of 0.43 ± 0.02. TALLYHO/JngJ mice (*n* = 3) showed an average Ct.Th of 0.22 ± 0.004 mm, Ct.BMD of 1134.7 ± 21.0 mg/cm^3^, and Ct.BV/TV of 0.94 ± 0.002.

**Figure 3:**
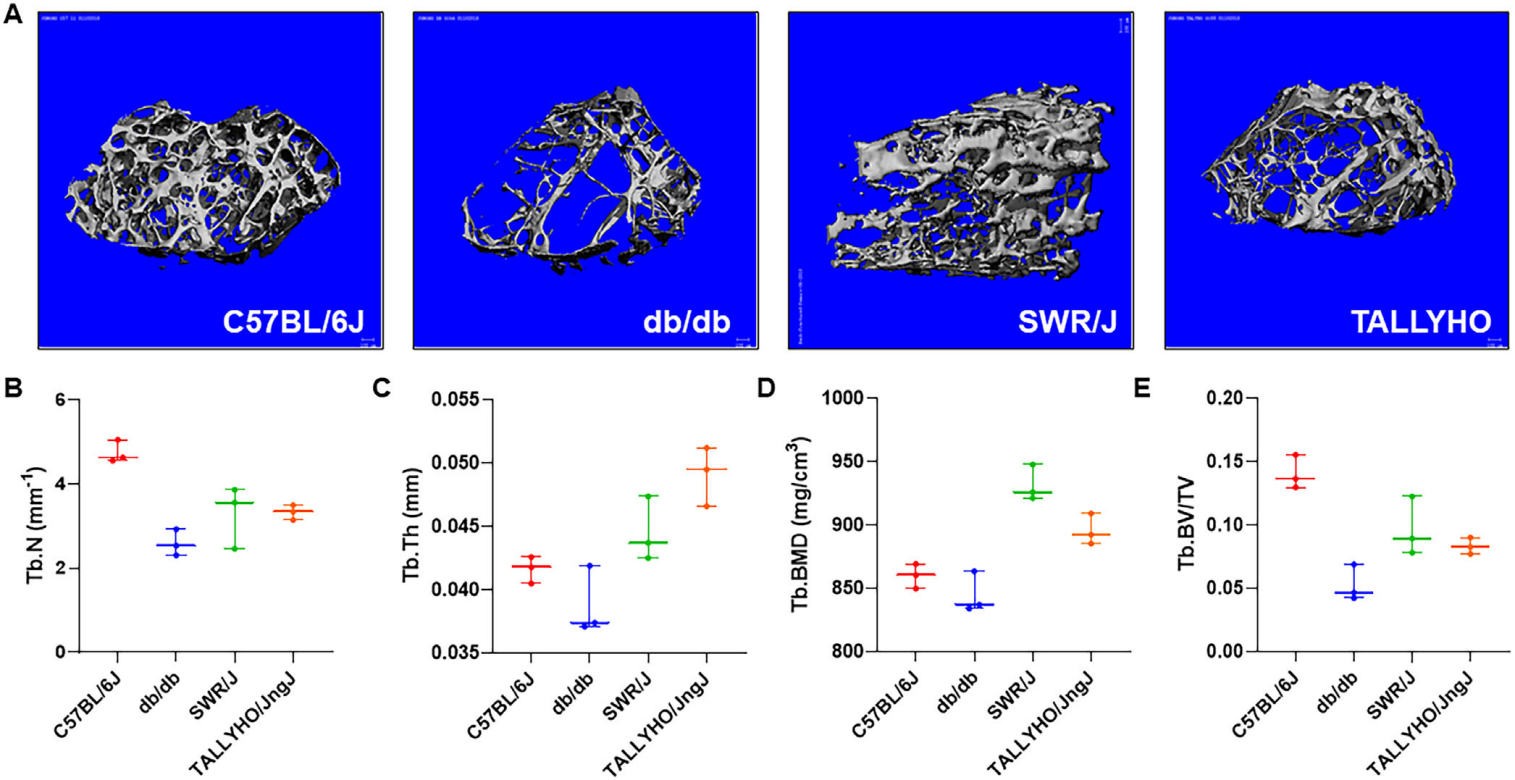
μCT analysis of femoral trabecular bone from type 2 diabetes mellitus murine models. (*A*) Representative images of the trabecular architecture of murine femurs. (*B–E*) Untreated femora from 10‐week old mice were analyzed using a Scanco μCT 35 system (Scanco Medical, Brüttisellen, Switzerland). C57BL/67 (*n* = 3) showed an average trabecular number (Tb.N) of 4.7 ± 0.3 mm^−1^, trabecular thickness (Tb.Th) of 0.04 ± 0.001 mm, trabecular bone mineral density (Tb.BMD) of 859.9 ± 9.7 mg/cm^3^, and trabecular bone volume fraction (Tb.BV/TV) of 0.14 ± 0.01. Db/db mice (*n* = 3) showed an average Tb.N of 2.6 ± 0.3 mm^−1^, Tb.Th of 0.04 ± 0.003 mm, Tb.BMD of 844.9 ± 16.2 mg/cm^3^, and Tb.BV/TV of 0.05 ± 0.01. SWR/J mice (*n* = 3) showed an average Tb.N of 3.3 ± 0.7 mm^−1^, Tb.Th of 0.04 ± 0.003 mm, Tb.BMD of 931.8 ± 14.1 mg/cm^3^, and Tb.BV/TV of 0.1 ± 0.02. TALLYHO/JngJ mice (*n* = 3) showed an average Tb.N of 3.3 ± 0.2 mm^−1^, Tb.Th of 0.05 ± 0.002 mm, Tb.BMD of 895.7 ± 12.3 mg/cm^3^, and Tb.BV/TV of 0.08 ± 0.01.

Untreated, harvested femora from 10‐week‐old mice from each strain were analyzed using μCT analysis to determine the characteristics of each strain's cortical Fig. 2 *A–D* and trabecular Fig. 3 *A–E* bone at baseline. μCT analysis of C57BL/6J mouse femoral cortical bone showed an average cortical thickness (Ct.Th) of 0.16 ± 0.01 mm, cortical bone mineral density (Ct.BMD) of 1028.7 ± 18.6 mg/cm^3^, and cortical bone volume fraction (Ct.BV/TV) of 0.91 ± 0.006. On analysis of db/db cortical bone, average Ct.Th was 0.14 ± 0.002 mm, Ct.BMD was 1057.4 ± 9.8 mg/cm^3^, and Ct.BV/TV was 0.90 ± 0.001. SWR/J cortical bone showed an average Ct.Th of 0.16 ± 0.007 mm, Ct.BMD of 1278.0 ± 5.6 mg/cm^3^, and Ct.BV/TV of 0.43 ± 0.02. Lastly, analysis of the cortical bone of TALLYHO/JngJ showed an average Ct.Th of 0.22 ± 0.004 mm, Ct.BMD of 1134.7 ± 21.0 mg/cm^3^, and Ct.BV/TV of 0.94 ± 0.002 Fig. 2 *B–D*.

μCT analysis of C57BL/6J mouse femoral trabecular bone showed an average trabecular number (Tb.N) of 4.7 ± 0.3 mm^−1^, trabecular thickness (Tb.Th) of 0.04 ± 0.001 mm, trabecular bone mineral density (Tb.BMD) of 859.9 ± 9.7 mg/cm^3^, and trabecular bone volume fraction (Tb.BV/TV) of 0.14 ± 0.01. Analysis of db/db trabecular bone showed an average Tb.N of 2.6 ± 0.3 mm^−1^, Tb.Th of 0.04 ± 0.003 mm, Tb.BMD of 844.9 ± 16.2 mg/cm^3^, and Tb.BV/TV of 0.05 ± 0.01. SWR/J trabecular bone analysis showed an average Tb.N of 3.3 ± 0.7 mm^−1^, Tb.Th of 0.04 ± 0.003 mm, Tb.BMD of 931.8 ± 14.1 mg/cm^3^, and Tb.BV/TV of 0.1 ± 0.02. TALLYHO/JngJ trabecular bone showed an average Tb.N of 3.3 ± 0.2 mm^−1^, Tb.Th of 0.05 ± 0.002 mm, Tb.BMD of 895.7 ± 12.3 mg/cm^3^, and Tb.BV/TV of 0.08 ± 0.01 Fig. 3 *B–E*.

### hPTH (1–34) increases cartilage development and CD51+ MSC proliferation in T2DM fracture calluses

**Figure 4:**
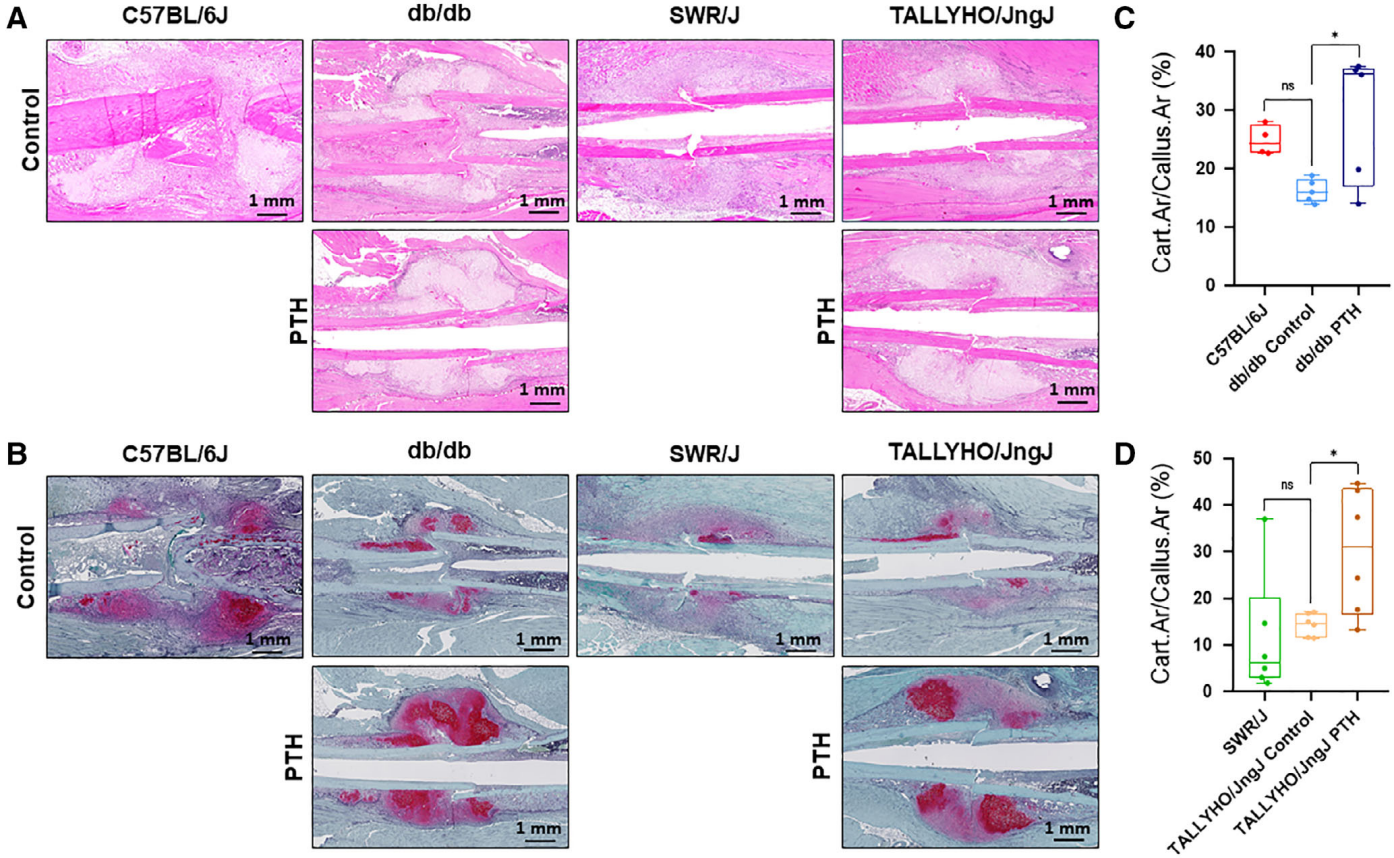
Safranin O staining for cartilage deposition in fracture callus. (*A*) Representative H&E histology of fracture calluses. (*B*) Representative safranin O staining of fracture calluses. (*C*,*D*) quantification of cartilage area in calluses from 10‐week‐old mice treated with 40 μg / kg hPTH (1–34) or without intervention are shown at day 7 postfracture. In untreated db/db mice, the average cartilage to callus area was 16.3 ± 2.0% (*n* = 5), whereas in db/db mice treated with hPTH (1–34) the average cartilage‐to‐callus area was 28.9 ± 11.1% (*n* = 5; *p* = 0.0274). C57BL/6J mice showed an average cartilage‐to‐callus area of 24.9 ± 2.6% (*n* = 4). Untreated TALLYHO/JngJ mice showed an average cartilage‐to‐callus area of 14.4 ± 2.4% (*n* = 6), whereas treated mice showed an increased average area of 30.1 ± 13.5% (*n* = 6; *p* = 0.0471). SWR/J mice showed an average cartilage‐to‐callus area of 11.6 ± 13.3% (*n* = 6). The difference between C57BL/6J and db/db control was not significant (*p* = 0.157). The difference between SWR/J and TALLYHO/JngJ control was not significant (*p* = 0.868). Red staining on safranin O denotes cartilage deposition. Measurements were performed using ImageJ software. ns = *p* ≥ 0.05, *p < 0.05; one‐way ANOVA.

**Figure 5:**
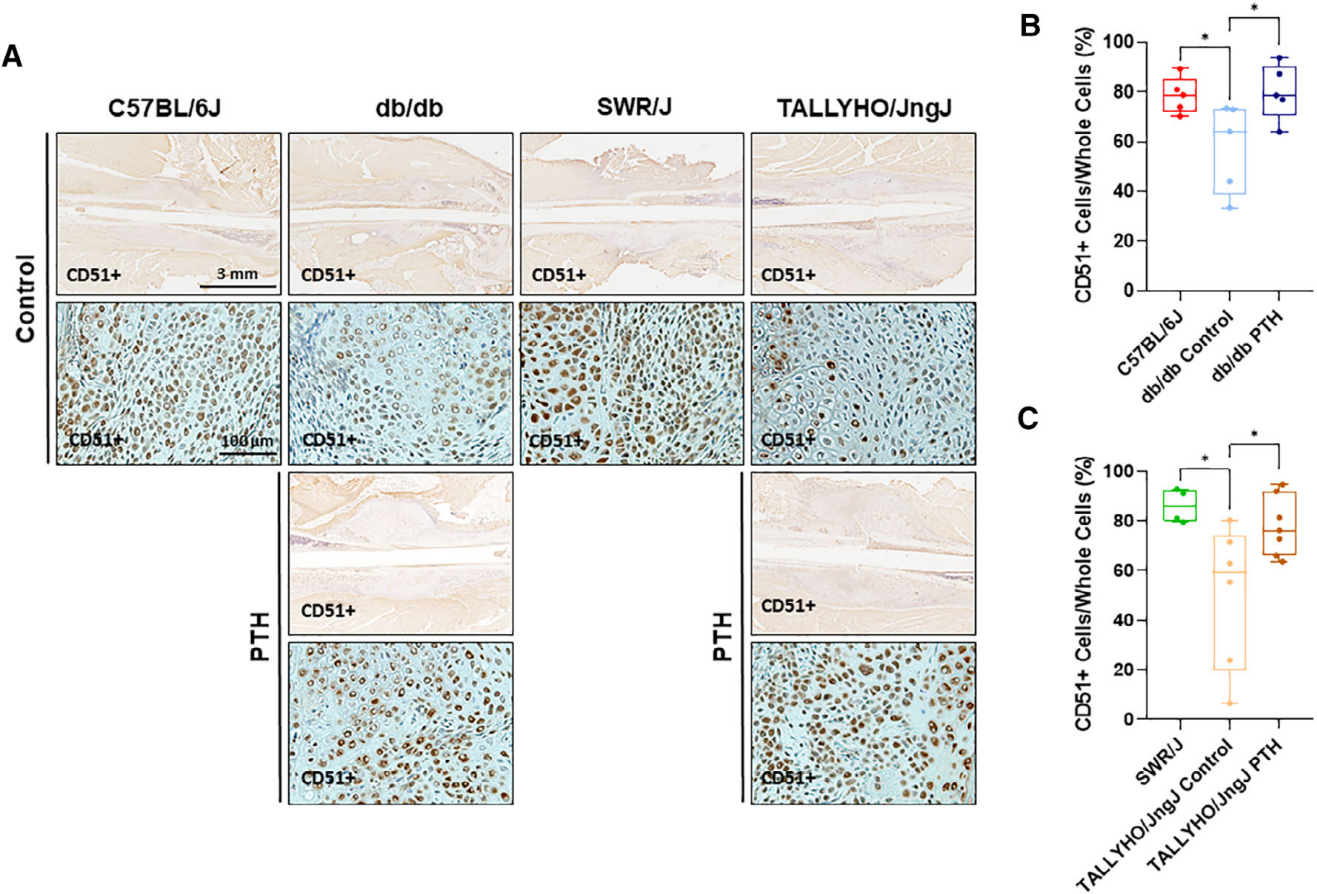
Immunohistochemistry for CD51+ MSC in fracture calluses. (*A*) Representative images of immunohistochemistry staining for CD51+ cells. (*B*,*C*) Quantification of immunohistochemistry analysis of callus formation in 10‐week‐old mice is shown at 7 days postfracture. C57BL/6J mice showed an average CD51+ percentage of 78.7 ± 7.3% (*n* = 5). Db/db mice treated with hPTH (1–34) showed an increase in average CD51+ cells to 80.1 ± 11.3% (*n* = 5) as compared with untreated db/db mice, which showed an average of 57.6 ± 18.0% (*n* = 5; *p* = 0.0353). SWR/J mice showed an average CD51+ percentage of 86.1 ± 6.8 (*n* = 4). TALLYHO/JngJ mice treated with hPTH (1–34) showed an increase in average CD51+ cells to 78.0 ± 12.0% (*n* = 7) as compared with untreated TALLYHO/JngJ mice, which showed an average 50.1 ± 28.8% (*n* = 6; *p* = 0.0374). The difference between C57BL/6J and db/db controls was significant (*p* = 0.0488) as was that between SWR/J and TALLYHO/JngJ controls (*p* = 0.0214). Yellow‐brown staining suggests a positive CD51 stain, a marker for mesenchymal stem cells. Measurements were performed using ImageJ software. *p < 0.05; one‐way ANOVA.

**Figure 6:**
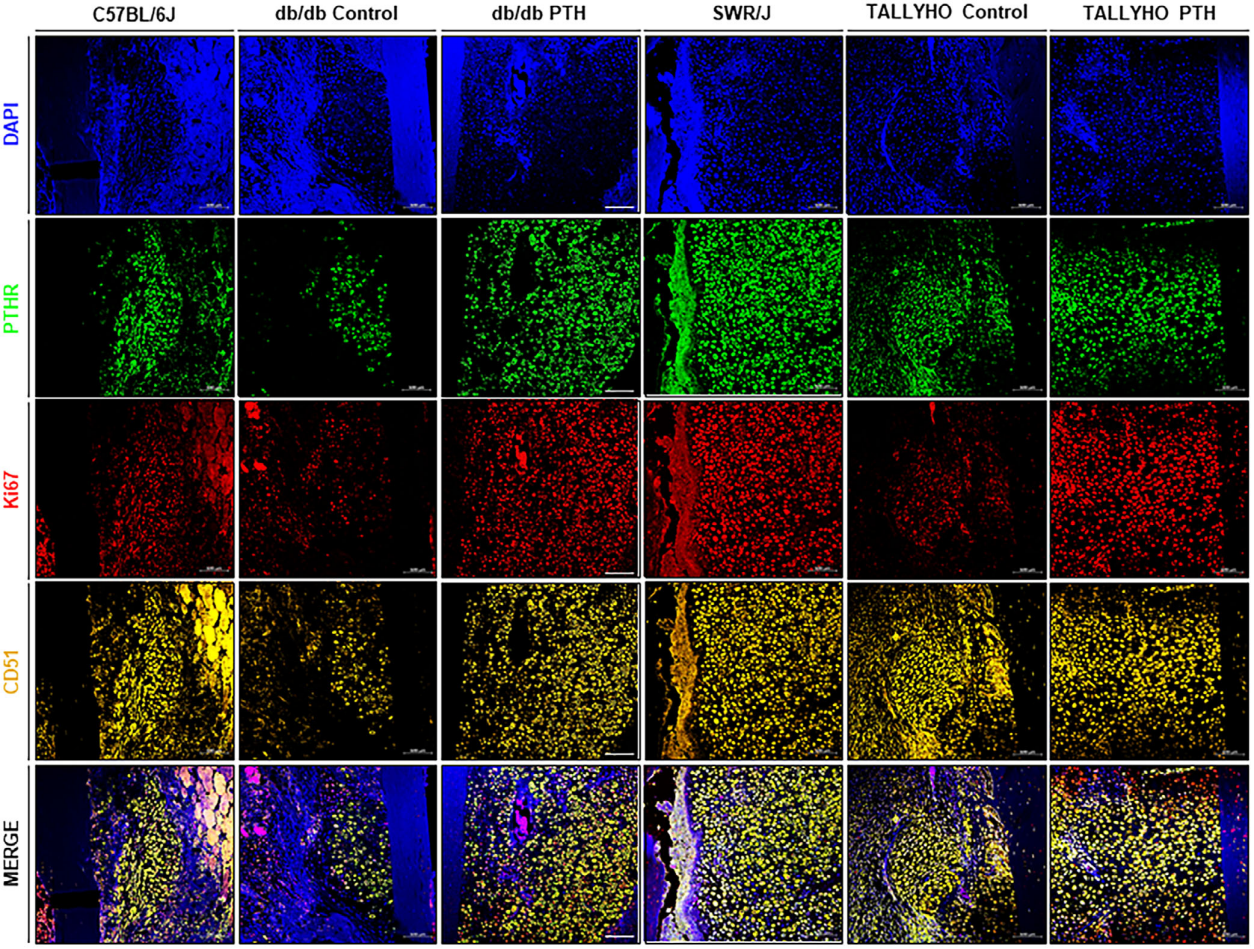
Multiplex immunohistochemistry staining for colocalization of PTHr1 and Ki67 in CD51+ mesenchymal stem cells (MSCs). Representative images of multiplex immunohistochemistry (m‐IHC) staining. Following quantification of IHC staining for CD51+, m‐IHC was performed to colocalize PTHr1 and Ki67. The membrane receptor PTHr1 colocalizes with the MSC membrane receptor CD51+; additionally, the nuclear protein Ki67 localizes to CD51+/PTHr1+ cells, which is demonstrative of accelerated proliferation in these cells. The data suggest that hPTH (1–34) increases MSC proliferation through a direct mechanism after interaction with PTHr1. Blue (4,6‐diamidino‐2‐phenylindole) stains for nuclei; green (fluorescein) stains for PTHr1; red (cyanine 5) stains for Ki67; orange (cyanine 3) stains for CD51.

Safranin O staining for cartilage revealed increased cartilaginous formation in fracture calluses in mice treated with hPTH (1–34) Fig. 4 *A–B*. In db/db mice, the average ratio of cartilage area to callus area was increased to 28.9% ± 11.1% for mice treated with hPTH (1–34) compared with 16.3% ± 2.0% in untreated mice (*p* = 0.0274); C57BL/6J mice showed an average cartilage‐to‐callus area ratio of 24.9% ± 2.6% Fig. 4 *C*. In TALLYHO/JngJ mice, this average ratio was 30.1% ± 13.5% in treated mice compared with 14.4% ± 2.4% in untreated mice (*p* = 0.0471); SWR/J showed an average cartilage‐to‐callus area ratio of 11.6% ± 13.3% Fig. 4 *D*. Moreover, IHC staining for CD51+ MSC proliferation in reclaimed calluses from T2DM mouse femora was performed 7 days postfracture; quantification of IHC staining showed increased proliferation of CD51+ MSC in both db/db and TALLYHO/JngJ mice treated with hPTH (1–34) Fig. 5 *A*. In db/db mice, the average ratio of CD51+ cells to total cells was 80.1% ± 11.3% for mice treated with hPTH (1–34) compared with 57.6% ± 18.0% in untreated db/db mice (*p* = 0.0353); C57BL/6J showed an average ratio of CD51+ cells to total cells of 78.7% ± 7.3 (Fig. 5 *B*). In TALLYHO/JngJ mice, this average ratio was 78.0% ± 12.0% for treated mice compared with 50.1% ± 28.8% in untreated mice (*p* = 0.0374); SWR/J showed an average ratio of CD51+ cells to total cells of 86.1% ± 6.8% (Fig. 5 *C*). Furthermore, m‐IHC showed colocalization of PTHr1 and Ki67 in CD51+ cells (Fig. 6).

### hPTH (1–34) administration increases bone mineralization in T2DM murine models

**Figure 7:**
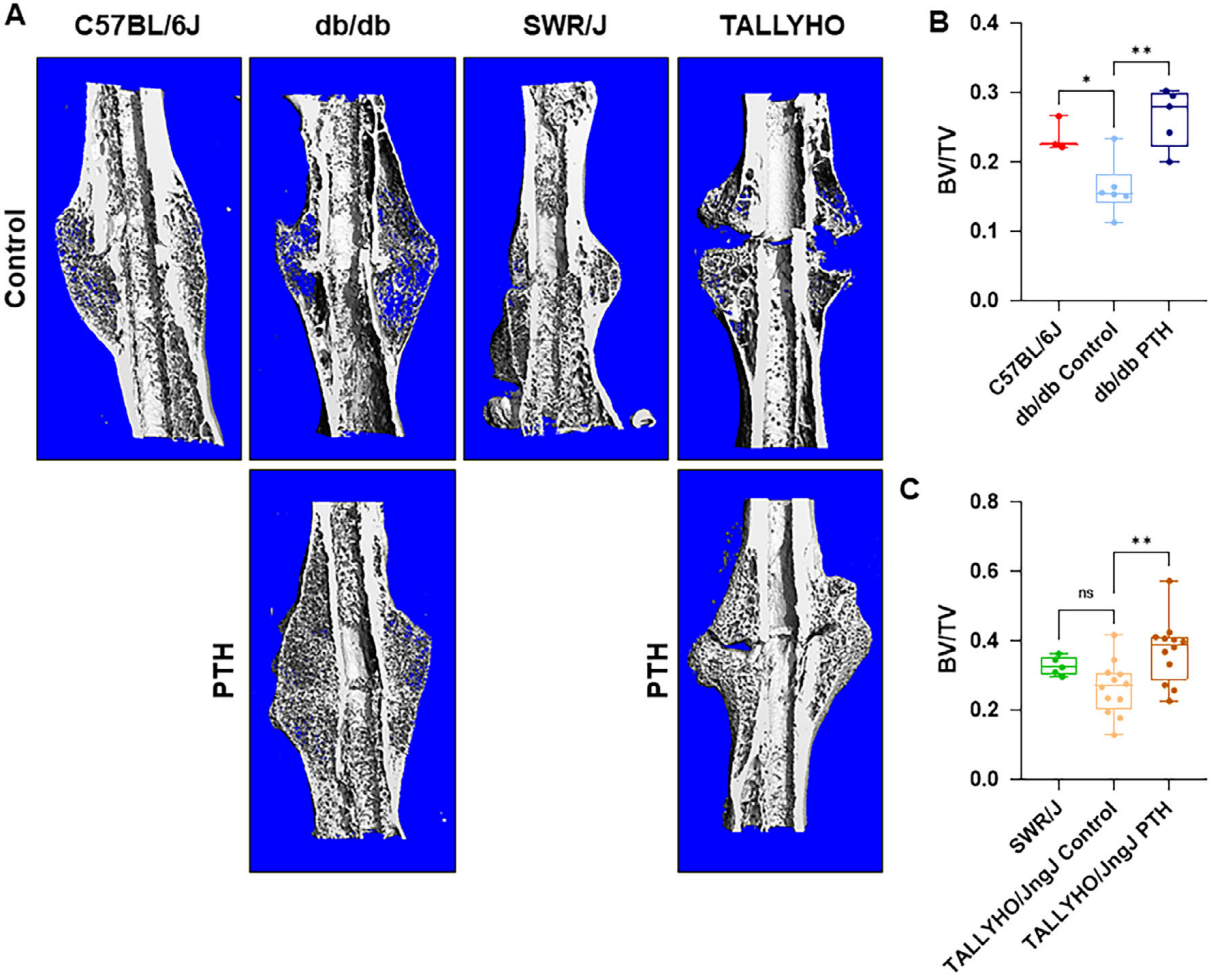
μCT analysis of fracture calluses. (*A*) Representative images of μCT‐scanned and graphically reconstructed global images of fracture calluses at 28 days postfracture. (*B*,*C*) Assessment of the ratio of bone volume to total volume (BV/TV) of healing femora from C57BL/6J (*n* = 3), db/db (Control *n* = 6, PTH *n* = 5), SWR/J (*n* = 5), and TALLYHO/JngJ mice (Control *n* = 12, PTH *n* = 12). Db/db and TALLYHO/JngJ mice treated with hPTH (1–34) showed a significantly increased average fraction of mineralized bone (BV/TV) at 0.25 ± 0.05 (*p* = 0.0020) and 0.37 ± 0.09 (*p* = 0.0054), respectively. This is compared with untreated db/db and TALLYHO/JngJ mice groups at 0.13 ± 0.03 and 0.26 ± 0.08, respectively. C57BL/6J and SWR/J mice showed an average fraction of mineralized bone of 0.24 ± 0.02 and 0.33 ± 0.03, respectively. The difference between C57BL/6J and db/db control was significant (*p* = 0.0326), whereas SWR/J and TALLYHO/JngJ controls were not (*p* = 0.254). ns = *p* ≥ 0.05, *p < 0.05, **p < 0.01; one‐way ANOVA.

μCT analysis of femora reclaimed from db/db and TALLYHO/JngJ mice treated with hPTH (1–34) for 28 days postfracture showed that hPTH (1–34) administration promoted fracture healing in diabetic mice Fig. 7 *A*. In femora reclaimed from diabetic mice, fracture calluses were appreciably smaller in size and showed more porous hard callus formation in untreated mice compared with calluses from fractured femora in mice treated with hPTH (1–34). Furthermore, db/db and TALLYHO/JngJ mice treated with hPTH (1–34) showed an increased fraction of mineralized bone (BV/TV) in healing calluses (Fig. 7). In db/db mice, average BV/TV was 0.25 ± 0.05 in mice treated with hPTH (1–34) compared with 0.13 ± 0.03 in untreated db/db mice (*p* = 0.0020); C57BL/6J showed an average BV/TV of 0.24% ± 0.02% (Fig. 7 *B*). In TALLYHO/JngJ mice, average BV/TV was 0.37% ± 0.09% in treated mice compared with 0.26 ± 0.08% in untreated TALLYHO/JngJ mice (*p* = 0.0054); SWR/J showed an average BV/TV of 0.33% ± 0.03% (Fig. 7 *C*).

### hPTH (1–34) administration increases force required to induce a repeat fracture

**Figure 8:**
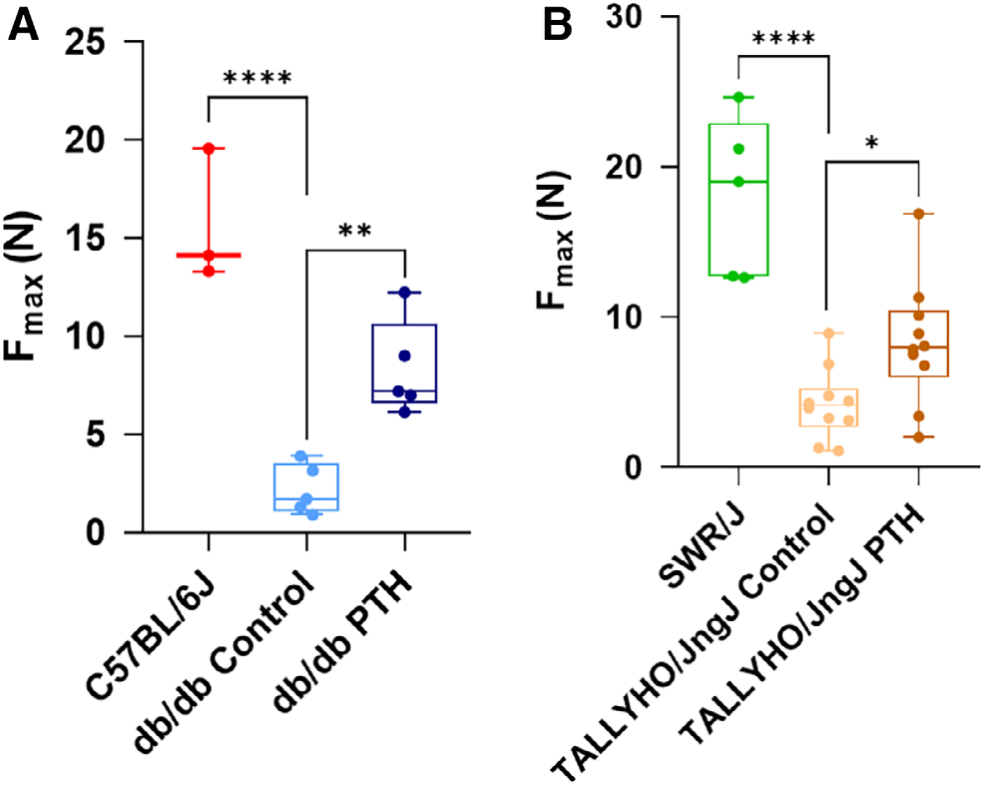
Three‐point mechanical stress testing of healed femora. (*A*,*B*) Quantification of mechanical stress testing results of mouse femora harvested 28 days after fracture induction with and without daily injections of hPTH (1–34). Db/db mouse femora treated with hPTH (1–34) required an average F_max_ of 8.3 ± 2.5 N (*n* = 5; *p* = 0.0034) to induce a fracture as compared with control db/db mice, which required an average of 2.2 ± 1.3 N (*n* = 5). C57BL/6J mice required an average of 15.7 ± 3.4 N (*n* = 3) to induce fracture. hPTH (1–34) treated TALLYHO/JngJ mouse femora required an average F_max_ of 8.3 ± 4.1 N (*n* = 10; *p* = 0.0456) as compared with their control counterparts, which required an average of 4.2 ± 2.4 N (*n* = 10) to induce a fracture. SWR/J mice required an average of 18.0 ± 5.3 N (*n* = 5) to induce fracture. The difference between C57BL/6J and db/db controls was significant (*p* = 0.0001). The difference between SWR/J and TALLYHO/JngJ controls was also significant (*p* = 0.0001). *p < 0.05, **p < 0.01; ****p < 0.0001; one‐way ANOVA.

Three‐point mechanical strength testing (MST) of femora reclaimed from db/db and TALLYHO/JngJ treated with hPTH (1–34) showed an increase in the maximal load (F_max_) required to induce a new fracture following initial fracture healing (Fig. 8). Db/db mouse femora treated with hPTH (1–34) required a significantly increased average F_max_ of 8.3 ± 2.5 N to induce a fracture as compared with control db/db mice, which required an average of 2.2 ± 1.3 N (*p* = 0.0034); C57BL/6J required an average of 15.7 ± 3.4 N to induce fracture (Fig. 8 *A*). hPTH (1–34) treated TALLYHO/JngJ mouse femora required an average F_max_ of 8.3 ± 4.1 N as compared with their control counterparts, which required an average of 4.2 ± 2.4 N to induce a fracture (*p* = 0.0456); SWR/J required an average of 18.0 ± 5.3 N to induce a new fracture (Fig. 8 *B*).

## Discussion

### TALLYHO/JngJ murine models of T2DM best mimic that of the human skeletal phenotype in T2DM

In this study, the effects of hPTH (1–34) on fracture healing in T2DM were tested. Currently, the intermittent systemic administration of hPTH (1–34) is utilized to treat senile osteoporosis,^21^, ^22^ osteoporosis secondary to prolonged glucocorticoid administration,^23^, ^24^, ^25^ and bisphosphonate‐associated osteonecrosis of the jaw.^26^, ^27^, ^28^, ^29^ Based on prior literature establishing that T2DM patients have both an increased risk of fracture^30^, ^31^, ^32^, ^33^, ^34^ and decreased PTH levels,^19^, ^35^, ^36^ it was determined practical to utilize hPTH (1–34) in the treatment of bone fractures secondary to T2DM. Therefore, the hypothesis of the present study was that hPTH (1–34) would improve the decreased bone‐quality phenotype and subsequently decrease the risk of bone fractures present in T2DM. To test this hypothesis, the determination of a relevant animal model was necessary. Given the complexities and polygenic etiology of T2DM in humans, there is currently no singular animal model of human T2DM that recapitulates the varied phenotypic presentations of the disease. Moreover, there is no consensus on the best murine model to study skeletal fragility secondary to T2DM. Therefore, two murine models of T2DM were analyzed: a single gene knockout murine model [B6.BKS(D)‐Lepr^db^/J (db/db)] and a polygenic murine model (TALLYHO/JngJ).

The db/db mouse is produced by a single gene mutation in the leptin receptor on the C57BL/6J background that results in extreme hyperphagia, subsequent morbid obesity, marked hyperglycemia, a compensatory hyperinsulinemia, and the resultant expansion of pancreatic β‐cell mass^37^; the data of the present study reiterated the obesity and hyperglycemia present in this strain. Furthermore, although db/db mice have elevated body mass and adiposity, which generally serves to increase bone mass,^38^, ^39^, ^40^ db/db mice have decreased bone volume (BV/TV) in both cortical and trabecular bone^41^—a fact recapitulated by the data of the present study. Additionally, prior studies have utilized histology to establish that db/db mice have decreased Ct.Th, Tb.N, and Tb.Th in long bones^42^; all of these metrics were verified in the present study. Moreover, Takeshita and colleagues determined that BMD of cortical and trabecular bone is decreased in db/db mice after 12 weeks of age, but not prior to this age range^43^; however, the mice used in this study were not within this age range and had normal to high BMD. It is well‐known that the bone of db/db mice has increased susceptibility to fractures given these factors.^44^

On the other hand, the TALLYHO/JngJ mouse is a polygenic murine model of T2DM that is characterized by increased body weight, moderate hyperglycemia, and hyperinsulinemia^45^; obesity and hyperglycemia were recapitulated in this study. Like all polygenic strains, TALLYHO/JngJ has no exact genetic control strains^45^, ^46^; TALLYHO/JngJ seems to be Swiss‐derived and shares 86.8% genotype homology with SWR/J, which is most often utilized as a control.^45^ Additionally, Devlin and colleagues showed that TALLYHO/JngJ mice have increased Ct.Th and decreased BV/TV, which this study verified; however, Devlin and colleagues showed more severe deficits in BV/TV than the present study and showed decreased Tb.N and Tb.Th in comparison to this study. BMD was also determined to be decreased in TALLYHO/JngJ by Devlin and colleagues, which was reiterated in the present study.^47^ The differences in the skeletal fragility phenotypes of TALLYHO/JngJ between the present study and the established literature are likely because of known discrepancies in the phenotypes of the strain based on acquisition location and environmental factors.^45^

With respect to humans, it is well‐known that although BMD is normal or increased in T2DM, patients are, paradoxically, at risk of increased bone fractures.^7^, ^32^, ^48^, ^49^, ^50^ However, the remainder of the literature regarding skeletal fragility in human T2DM shows vastly varied findings with respect to cortical and trabecular bone compartments.^51^, ^52^, ^53^, ^54^ Given these facts, we suggest TALLYHO/JngJ is the best model for the study of skeletal fragility in T2DM and fracture healing because of its polygenic background much like that of the human disease and increases in cortical bone compartment. Furthermore, db/db mice have deficiencies in the cortical and trabecular compartments that are different from and do not explicitly fit the human phenotype. Moreover, leptin is responsible for reducing bone fragility by inducing osteoblast proliferation and inhibiting osteoclastogenesis.^55^ Therefore, the skeletal fragility found in db/db mice is likely primarily caused by the loss of leptin's effects, which does not mechanistically mimic the proinflammatory state of diabetes that induces osteoclastogenesis and the subsequent skeletal fragility found in diabetic humans.^56^

### hPTH (1–34) rescues bone fracture healing in murine models of T2DM bone fragility

Multiple studies in humans^57^, ^58^, ^59^ and animal models^60^, ^61^, ^62^, ^63^ have determined the benefits of systemic recombinant human PTH in fracture healing and age‐related osteoporosis. In Sprague–Dawley rats, Holzer and colleagues showed that hPTH (1–34), when administered intermittently, enhanced fracture‐healing^62^; utilizing the same rat strain, this fact was recapitulated by Alkhiary and colleagues^(60)^ and Nakajima and colleagues.^63^ However, literature concerning the effects of hPTH (1–34) in the T2DM state is relatively limited. Indeed, Liu and colleagues is the only study, to our knowledge, presently available that studies the effects of fracture healing in diabetic rodents and how to rescue the phenotype: They were able to show that exogenous PTH‐related peptide was able to accelerate fracture healing in db/db mice.^64^ Conversely, Ohuchi and colleagues showed that hPTH (1–34) improved BMD and bone strength in Akita diabetic mice, but did not test fracture healing.^65^ Suzuki and colleagues also tested the effects of hPTH (1–34) on streptozotocin‐induced diabetic Wistar rats and found that the treatment increased BMD, but failed to test fracture healing as well.^66^ Therefore, the present study represents one of the few studies concerning systemic hPTH (1–34) administration in diabetic fractures and explores untested rodent strains. Based on histological, μCT, and mechanical stress testing data, intermittent systemic hPTH (1–34) improves the delayed fracture‐healing phenotype of T2DM.

Indirect fracture healing recapitulates both the endochondral and intramembranous ossification that are paramount in embryological skeletal formation.^67^ Indirect fracture healing progresses through four distinct stages: hematoma formation, generation of a cartilaginous callus (soft callus), resorption of cartilage with mineralization of the callus (hard callus), and bone remodeling.^67^ Ultimately, the endochondral ossification required in indirect fracture healing is dependent on the mineralization of a soft callus; therefore, the greater the deposition of cartilage in the soft callus, the larger the callus and more accelerated and robust the ultimate fracture healing. Previous literature, however, has elucidated the fact that fracture calluses in diabetics are significantly smaller in size as compared with those generated in a normal metabolic state.^68^, ^69^, ^70^, ^71^ The present study shows that intermittent systemic hPTH (1–34) increases cartilage formation in and size of the soft callus at 7 days postfracture, the peak of soft callus formation in animal models.^72^ Moreover, Ko and colleagues showed that the excessive production of inflammatory cytokines that is pathognomonic of the diabetic state, specifically increased expression of TNFα, reduces MSC numbers in fracture callus areas by decreasing proliferation and increasing the apoptotic rate of MSCs.^73^ The present study shows that intermittent systemic hPTH (1–34) administration ameliorates the decreased number of CD51+ MSCs in the chosen diabetic murine models. Furthermore, multiplex immunohistochemistry shows that CD51+ MSC coexpress membrane PTHr1 and the nuclear marker of cell proliferation, Ki67, suggesting that hPTH (1–34) is directly responsible for restoring MSC proliferative capacity in T2DM mouse models.

Previous studies have shown that the fracture callus mineralization—development of the hard fracture callus—is reduced and delayed in diabetes.^68^, ^69^, ^74^ Likewise, patients with T2DM have been shown to have decreased mechanical strength of bone following fracture healing.^70^, ^71^, ^75^ Therefore, the diabetic state not only attenuates early fracture healing by decreasing cartilaginous callus generation, it also decreases the mineralization and formation of new bone in late fracture healing. Data from the present study shows that treatment with hPTH (1–34) increases the BV/TV in fracture calluses at day 28, the peak of mineralization and beginning of woven bone deposition in rodent models^72^; these data suggest reversal of the delayed late fracture healing phenotype found in T2DM. Furthermore, data collected on mechanical stress testing conducted during this study illustrate that an increased amount of force is required to induce a repeat fracture after completion of bone fracture healing in the chosen murine models. Increased F_max_ to induce a fracture is demonstrative of further increased callus mineralization and bone formation. Therefore, it is evident that hPTH (1–34) treatment increases both early and late fracture healing in a mouse model with T2DM. hPTH (1–34) shows promise in reversing the negative sequelae of T2DM on bone fracture healing; however, clinical trials are needed moving forward to assess the replicability of our findings and efficacy of treatment in a human model.

## Disclosure

MDR is a consultant for Paragon 28 Inc. No other author has a conflict of interest.
